# A Health Economic Evaluation of Routine Hepatocellular Carcinoma Surveillance for People with Compensated Cirrhosis to Support Australian Clinical Guidelines

**DOI:** 10.1177/23814683251344962

**Published:** 2025-06-26

**Authors:** Joachim Worthington, Emily He, Michael Caruana, Stephen Wade, Barbara de Graaff, Anh Le Tuan Nguyen, Jacob George, Karen Canfell, Eleonora Feletto

**Affiliations:** The Daffodil Centre, The University of Sydney, a joint venture with Cancer Council NSW, Sydney, NSW, Australia; The Daffodil Centre, The University of Sydney, a joint venture with Cancer Council NSW, Sydney, NSW, Australia; School of Public Health, Faculty of Medicine and Health, University of Sydney, Sydney, NSW, Australia; The Daffodil Centre, The University of Sydney, a joint venture with Cancer Council NSW, Sydney, NSW, Australia; Menzies Institute for Medical Research, The University of Tasmania, Hobart, Tasmania; Menzies Institute for Medical Research, The University of Tasmania, Hobart, Tasmania; WHO Collaborating Centre for Viral Hepatitis, The Peter Doherty Institute for Infection and Immunity; Storr Liver Centre, The Westmead Institute for Medical Research, Westmead Hospital and University of Sydney, Sydney, NSW, Australia; School of Public Health, Faculty of Medicine and Health, University of Sydney, Sydney, NSW, Australia; The Daffodil Centre, The University of Sydney, a joint venture with Cancer Council NSW, Sydney, NSW, Australia

**Keywords:** cancer screening, health economics, hepatocellular cancer, liver cancer, mathematical model, public health, secondary prevention

## Abstract

**Background.** Liver cancer is the only cancer in Australia with rising incidence and mortality rates, despite the potential for early detection through surveillance of high-risk individuals. Hepatocellular carcinoma (HCC), the most common form of primary liver cancer, has curative treatment options available if detected early. Six-monthly HCC surveillance is recommended for people with liver cirrhosis and was proposed for inclusion in the 2023 Cancer Council Australia *Clinical Practice Guidelines for Hepatocellular Carcinoma Surveillance for People at High Risk in Australia*. To evaluate the proposed 2023 guideline recommendation, we developed Policy1-Liver, a novel mathematical model of liver disease, HCC, and surveillance. We then assessed the health and economic implications of 6-monthly HCC surveillance in Australia via ultrasound, with or without alpha-fetoprotein. **Methods.** Policy1-Liver was calibrated to existing data sources on liver disease, HCC, and health care costs in Australia. We assessed the impact of 6-monthly routine HCC surveillance with ultrasound with or without alpha-fetoprotein testing as well as a range of other sensitivity analyses and alternative scenarios such as varying surveillance adherence and intervals to assess potential future modifications to surveillance. **Results.** We estimated that 6-monthly HCC surveillance, with or without alpha-fetoprotein, can increase early-stage diagnoses to up to 81% and reduce HCC mortality by 22% in people with cirrhosis. We estimate an incremental cost-effectiveness ratio of $28,423 per quality-adjusted life-year for 6-monthly surveillance with ultrasound alone compared with no surveillance. **Conclusions.** These findings support guideline-recommended 6-monthly HCC surveillance with ultrasound, affirming its health benefits and cost-effectiveness, and demonstrate the potential to improve cost-effectiveness by refining surveillance intervals and improving early-stage HCC survival. Supporting implementation of the surveillance guidelines will play a key role in improving HCC mortality rates in Australia.

**Highlights:**

- Routine surveillance can improve the likelihood of early-stage detection of liver cancer, improving survival.
- Our modeling found that routine HCC surveillance with ultrasound would be cost-effective for people with liver cirrhosis in Australia.
- These findings can inform guidelines and investment in liver cancer control for high-risk patients.

## Graphical abstract

**Graphical Abstract:**
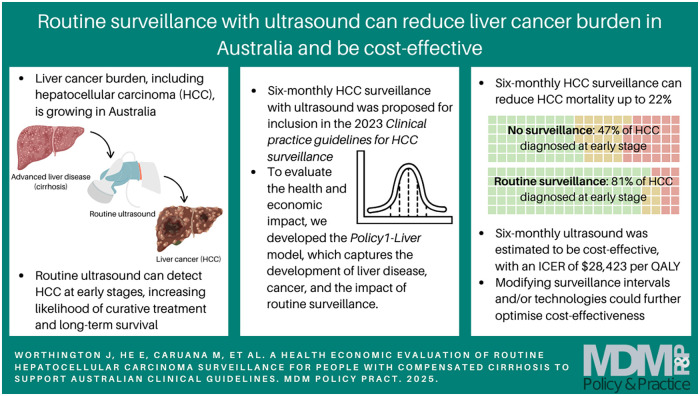
This is a visual representation of the abstract.

Worldwide, liver cancer is the fourth most common cause of cancer death^1^; in Australia, it is the seventh most common cause of cancer-related death,^2^ driven by increasing trends in liver cancer incidence, with deaths projected to double by 2040.^3^ A key step to improving mortality outcomes is increased detection at early stages, to improve the potential for curative treatment.^4–6^

Hepatocellular carcinoma (HCC), the most common form of primary liver cancer, can be detected early through routine HCC surveillance, which is typically recommended for high-risk populations.^7^ Previous Australian^8,9^ and international^10–14^ clinical guidelines and consensus statements recommend HCC surveillance for people at high risk, primarily those who have already developed cirrhosis (late-stage liver disease). Despite these recommendations, few people diagnosed with HCC have received any routine surveillance,^15^ and among those who had, adherence was limited.^16^ More evidence is needed to evaluate and improve HCC surveillance recommendations and support their implementation in Australia.^7^

To address this, the 2023 *Clinical Practice Guidelines for HCC Surveillance for People at High Risk in Australia*^8^ (hereafter *HCC surveillance guidelines*) were developed to provide cohesive recommendations for the management of people at high risk of HCC in Australia.^8,17^ The guidelines were led by a multidisciplinary working party, including health care and clinical representatives, representatives with lived experience, and other community representatives. The HCC surveillance guidelines included recommendations for routine HCC surveillance via 6-monthly ultrasound (US) imaging for people with compensated liver cirrhosis, with the potential addition of alpha-fetoprotein (AFP) blood testing.

To support this recommendation, it was necessary to estimate the health and economic impacts of surveillance. Assessing the recommendations poses significant challenges, as it requires evaluating and balancing the health benefits against long-term costs^18^ and identifying optimal technologies and intervals,^19^ and it can be limited by barriers to adherence.^20^ Understanding these is necessary to develop an economic case for health system investment, especially given calls for a national screening program in Australia.^21^ However, large randomized control trials of HCC surveillance are not feasible.^22^

Health economic modeling can fill evidence gaps where trials are not possible or feasible, generating estimates of the benefits, costs, and resource utilization of interventions. In Australia, modeling has been used to support screening and surveillance recommendations across cancers including bowel, cervical, and lung cancer.^23–26^ Internationally, health economic modeling studies have analyzed liver cancer control,^7,8,27^ and Australian modeling has estimated that 6-monthly US is likely cost-effective for patients with cirrhosis^18^ and that biomarker testing can stratify risk to improve cost-effectiveness.^28^ However, economic models of HCC and surveillance are less established in Australia than for other cancers.^23^ The accuracy and the precision of HCC surveillance modeling can be improved significantly by capturing detailed data on the progression of liver disease, incorporating detailed survival data, and operating at flexible time scales to evaluate and refine surveillance recommendations, which is not possible in Markov models. Capturing this level of detail, particularly around the development of cirrhosis and competing risk of decompensation, has been identified as a priority in liver cancer modeling.^29^

To address this need, we developed Policy1-Liver, a new model of liver disease, HCC, and surveillance. We used the novel sojourn time density framework to develop the model,^30^ which can incorporate complex epidemiological processes while remaining fast enough to perform robust sensitivity analyses. We calibrated Policy1-Liver to relevant existing data sources on the development of liver disease and cancer. We then used Policy1-Liver to evaluate the health and economic implications of the 2023 guideline recommendations for 6-monthly HCC surveillance in Australia via US, with or without AFP.

Further analyses were also completed to assess the sensitivity of the model assumptions and the potential cost-effectiveness of modifications to the surveillance recommendations. These included estimates of the impact of alternative surveillance intervals, patient age at cirrhosis diagnosis, patient adherence to surveillance recommendations, and alternative HCC treatment costs and effectiveness.

## Methods

**Figure 1:**
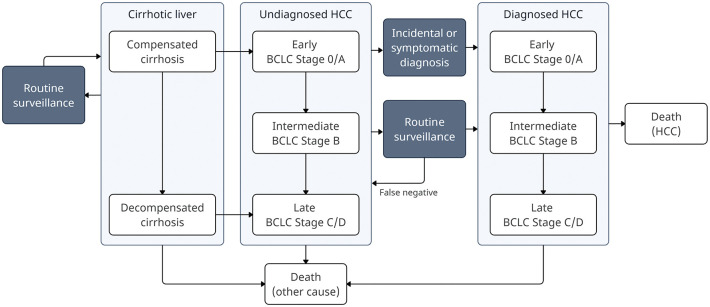
A simplified schematic of the key states of the model of HCC and liver surveillance.

We developed the Policy1-Liver health economic model of liver disease, HCC, and surveillance for our analyses. The structure of the model is illustrated in Figure 1. The design and development of the model was conducted in parallel to the development of the HCC surveillance guidelines. The guidelines working group informed the design of the model, the scenarios analyzed, and the aims of this economic analysis. The model was developed according to the Consolidated Health Economic Evaluation Reporting Standards (CHEERS) checklist, which is included in Appendix C: CHEERS Checklist.^31^

Policy1-Liver was developed using the sojourn density model framework.^30^ This novel mathematical approach to epidemiological modeling computes the evolving probability distribution of a patient being in a particular state at any given time as well as the time spent in that state (the sojourn time), based on sojourn-time–dependent hazard rates. These hazard rates encode the likelihood of transitioning from one health state to another based on the year, patient age, and time spent within the current health state. The sojourn density model framework captures a high level of detail while being quick and deterministic to avoid high computational burden and allow large-scale sensitivity analyses.

Parameters were directly derived or calibrated from relevant sources identified in a review completed for the HCC surveillance guidelines, with Australian studies used where possible. Where Australian trial data were not available, data from meta-analyses with large cohort sizes were prioritized. The full list of sources and calibration targets for the model parameters are included in Appendix A, Appendix Tables 1–6.

The model estimates disease risk from time of diagnosis of compensated liver cirrhosis, which can develop into decompensated cirrhosis and/or undiagnosed HCC. These states were selected to capture the most clinically relevant details based on the available data. HCC is diagnosed either symptomatically/incidentally or at a routine HCC surveillance event. The Australian liver cirrhosis patient population was modeled for the baseline analysis, based on age distribution and mortality rates. Decompensation rates for patients with compensated liver cirrhosis were directly available from cohort studies.^32^

HCC incidence, diagnosis, and upstaging rates were calibrated based on time-to-event data for HCC diagnosis and observed stage at diagnosis.^32,33^ This was supplemented with data on observed stage at diagnosis in cohorts routinely undergoing HCC surveillance^33^ and data on the sensitivity and specificity of HCC surveillance.^34^ Parametric forms for all hazard rates were chosen to best fit the data while minimizing the number of parameters to avoid potential overfitting. Where necessary, the Nelder–Mead algorithm was used to determine the best-fit parameters by minimizing the mean square error between the model outputs and the target data. To assess the model’s sensitivity to uncertainty in the key model parameters, 1-way sensitivity analyses was completed on the natural history parameters.

HCC stage at diagnosis was modeled according to the Barcelona Clinic Liver Cancer (BCLC) staging system,^35^ which is based on a patient’s Eastern Cooperative Oncology Group (ECOG) performance status,^36^ Child–Pugh score,^37^ and tumor stage. Cancers were identified as early (BCLC stage 0/A: ECOG status 0, tumor ≤ 3 cm), intermediate (BCLC stage B: ECOG status 0, multinodular), or late stage (BCLC stage C/D: ECOG status ≥ 1, vascular invasion, extrahepatic spread, and/or Child–Pugh C). These groupings were chosen to align with available survival data. Patients with compensated cirrhosis initially develop early-stage HCC, which can subsequently progress; patients with decompensated cirrhosis develop late-stage HCC only. The modeled stage is a stage at diagnosis only; postdiagnosis progression is not explicitly modeled but is captured in modeling of stage-at-diagnosis survival per the available data.

The diagnosis to confirm suspected HCC, either after symptomatic development or positive surveillance event (including false positives), was modeled as computed tomography or magnetic resonance imaging, with biopsy in cases in which imaging was insufficient.^18^ People who experience liver decompensation do not receive HCC surveillance, per recommendations.^7^

Five-year stage-specific overall HCC survival rates were calculated based on data from the NSW Cancer Registry, as these data are stratified by stage.^38^ This was augmented by national data.^39^ International studies were used to inform survival by time since diagnosis, stage, and screen versus symptomatically detected HCC.^40^ Health outcomes relating to treatment modalities, including serial treatments, were not modeled explicitly but were estimated to determine treatment costs (see below). HCC recurrence more than 5 y after initial diagnosis was not modeled explicitly due to lack of data; HCC recurrence within 5 y of the initial diagnosis was included in the modeled 5-y survival rates. Other-cause mortality was modeled for people with compensated or decompensated cirrhosis^41^ relative to the age-specific mortality rate in Australian data.^42^

The benefits of surveillance were measured in quality-adjusted life-years (QALYs) saved. For the quality-of-life estimates, health state utilities were identified from a study of patient preferences using standard gamble methods.^43^ Quality of life was stratified by liver cirrhosis or HCC and type of HCC treatment required.

Costs for surveillance and diagnostic procedures were collated from the Australian Medicare Benefits Schedule.^44^ Other costs included annual costs of cirrhosis care for patients with and without decompensation and end-of-life costs.^45,46^ Costs were analyzed from a health system perspective to capture the most relevant cost for Australian policy makers and presented in 2023 Australian dollars with costs adjusted using the Australian health Consumer Price Index.^47^ Costs relating to cancer treatment were based on an excess-cost study that stratified patients by their primary treatment,^48^ which varied by stage at diagnosis. These data were chosen as they are locally relevant and based on real-world observations rather than prescribed treatment recommendations. Cost inputs are included in Appendix A.

A cost-utility analysis was conducted by estimating the incremental cost-effectiveness ratio (ICER) as the cost per QALY saved associated with surveillance versus the previous most cost-effective intervention. A patient lifetime time horizon was included to capture the full downstream benefits of surveillance. For the cost-effectiveness analysis, a 5% annual discount rate was applied to both costs and QALYs, in line with other health economic analyses in Australia.^49^ A willingness-to-pay (WTP) threshold of $50,000/QALY is included as a reference for incremental cost-effectiveness, with interventions below this threshold considered cost-effective. This indicative threshold is based on previous analyses in the Australian context, although this is not an official threshold endorsed by the Australian Government Department of Health and Aged Care.

## Scenarios

### Main Analysis

For the main guidelines analysis, we estimated the health benefits and economic impacts of 6-monthly HCC surveillance with US, with or without AFP testing, versus no routine HCC surveillance as the comparator (status quo for people with cirrhosis in Australia^15^). This was modeled for a cohort with a mean age of 51 y (standard deviation 11 y) to reflect characteristics of the cirrhotic population,^50^ by aggregating single-age simulations. These surveillance scenarios were selected as part of the development of the HCC surveillance guidelines based on expert consultation with the working group and international recommendations.^8,9,13,17,51^ For this baseline scenario, patients were assumed to have perfect adherence to surveillance recommendations.

### Sensitivity Analyses

A probabilistic sensitivity analysis on the impact of 6-monthly US with or without AFP was completed to reflect the parameter uncertainty from the various data sources. Parameter values were sampled from the distributions listed in Appendix A. For each parameter set, the model was run to simulate outcomes with no surveillance and 6-monthly HCC surveillance through US alone or US with AFP. We simulated 100,000 parameter sets, with the most incrementally cost-effective option identified for each set. To assess the impact of potential future changes to HCC treatment technologies, we completed a 2-way sensitivity analysis on the parameters relating to HCC survival and treatment costs by stage. For each HCC stage, we analyzed the cost-effectiveness of 6-monthly US surveillance with 50% lower and 50% higher treatment costs and/or 50% lower and 50% higher 5-y survival. A further sensitivity analysis on the impact of the choice of discount rate was completed, as recommended by the Australian Government Department of Health and Aged Care and Medicines Australia.^52,53^

### Further Analyses

Additional analyses were simulated to assess potential areas for exploration in alternative surveillance recommendations. The first of these assessed the ICER for routine HCC surveillance by US with or without AFP testing at intervals of 1, 3, 6, 12, 24, and 48 months. The second additional analysis assessed the cost-effectiveness of surveillance by 6-monthly US by age of cirrhosis diagnosis (40, 41, 42, . . . , 75 y). The final analysis assessed the costs and health benefits HCC surveillance by 6-monthly US with or without AFP by adherence to surveillance recommendations (between 0% and 100%). These additional analyses were designed to assist with planning around future surveillance recommendations and provide suggestive evidence on potential improvements.

## Results

### Model Calibration

**Figure 2:**
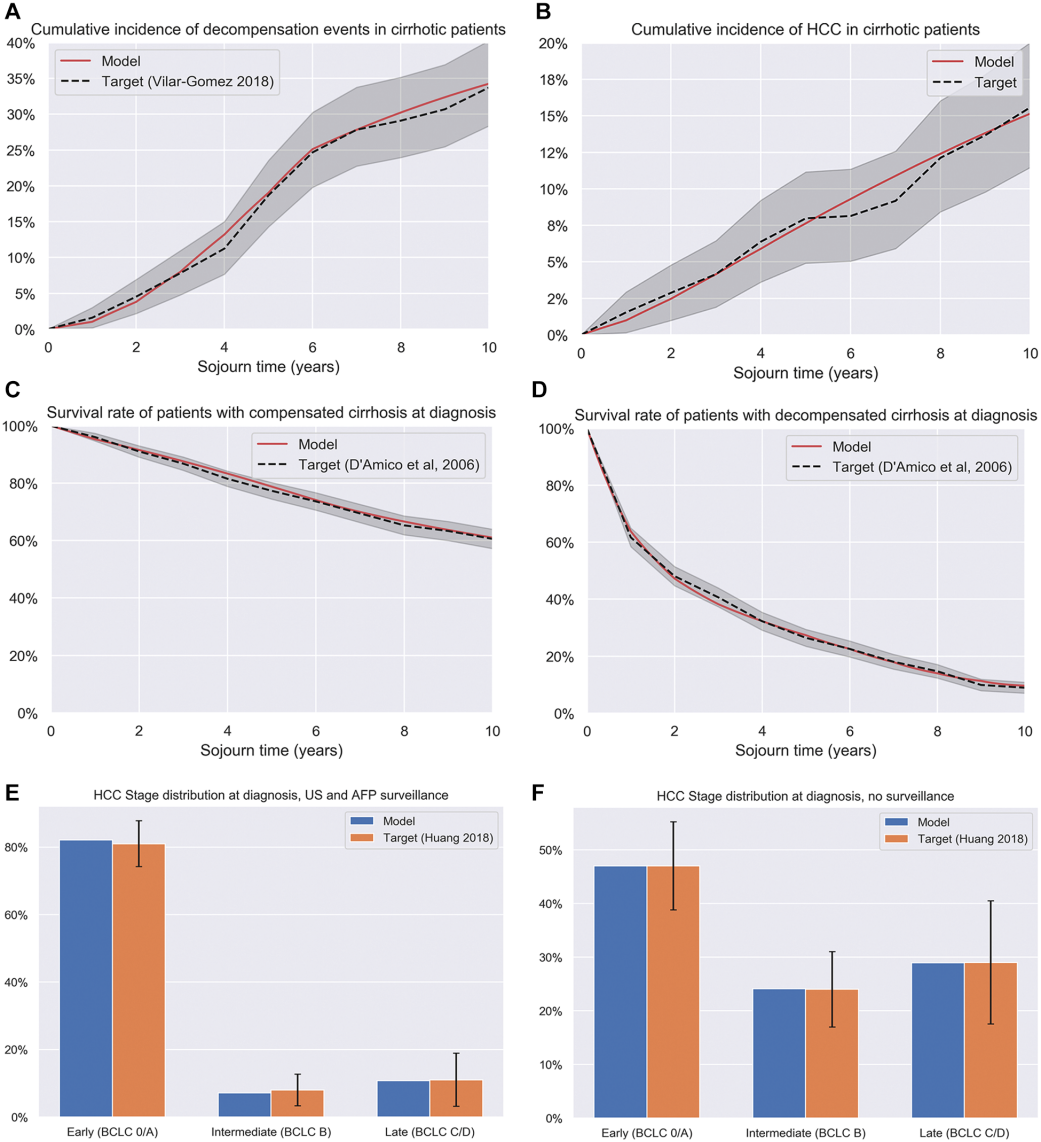
Selected calibration targets for the HCC model. AFP, alpha-fetoprotein; BCLC, Barcelona Clinic Liver Cancer; HCC, hhepatocellular carcinoma; US, ultrasound.

The model was calibrated to reproduce key targets and ensure the estimates were reliable. The model was successfully fitted to the target data, reproducing the terminal values for the survival curves and remaining within the 95% confidence intervals for the duration of the data (Figure 2) and reproducing values with the 95% confidence intervals for other targets. Key hazard rates are shown in Table 1, and the full parameter set for the model is shown in Appendix A, including calibration targets and parameter distributions used in the sensitivity analyses.

**Table 1:** Selected parameter values for the model, including hazard rates λ for state transitions.

Parameter	Value	Calibration Target
Cirrhosis decompensation hazard rate	$\lambda =\left\{ \begin{array}{c} 0.0204\tau +0.0106\;\mathrm{for}\;\tau <6\\ 0.0554\mathrm{for}\;\tau \geq 6 \end{array}\right.$	Vilar-Gomez et al.^32^
HCC development hazard rate	$\lambda =0.0102\tau^{1.377}$	
Death hazard rate (compensated cirrhosis)	$\lambda =0.0336\tau^{-0.2379}$	D’Amico et al.^41^
Death hazard rate (decompensated cirrhosis)	$\lambda ={\left(0.1539\tau -0.4865\right)}^{2}+0.2103$	
Stage 0/A progression hazard	λ= 0.2049	Huang et al.^33^
Stage B progression hazard	λ= 1.0259	
Stage 0/A progression hazard	λ= 0.2091	
Stage B progression hazard	λ= 1.1727	
Stage C/D progression hazard	λ= 1.4887	
Five year survival, stage 0/A/B	47.7%	NSW Cancer Registry.^38^
Five year survival, stage C/D	20.6%	
Survival HR, stage B v. stage 0/A	HR= 0.508	Haq et al.^40^
Survival HR, stage D v. stage C	HR= 0.841	
US sensitivity, stage 0/A HCC	53%	Tzartzeva et al.^34^
US sensitivity, stage B/C/D HCC	84%	
US specificity	91%	
US and AFP sensitivity, stage 0/A HCC	63%	
US and AFP sensitivity, stage B/C/D HCC	97%	
US and AFP specificity	84%	

AFP: alpha-fetoprotein; HCC, hepatocellular carcinoma; HR, hazard ratio; US, ultrasound.

τ is the sojourn time in state. Further details, including distributions for sensitivity analyses, are included in Appendix A.

#### Main analysis: 6-monthly HCC surveillance

The outcomes for 6-monthly HCC surveillance are shown in Table 2. Six-monthly HCC surveillance with US alone would reduce the likelihood of HCC death in a person with cirrhosis by 21.7% compared with no intervention, increasing the quality-adjusted life expectancy by 6.2%. Six-monthly US with the addition of AFP would reduce the likelihood of HCC death by 22.9%, increasing the quality-adjusted life expectancy by 6.5%. This is attributable to improvements in early-stage diagnosis; the probability of HCC diagnosed at early (BCLC A/0) stage would increase to 81.3% for those who undergo 6-monthly US surveillance with AFP compared with 47.0% in the cohort with no surveillance. On average, people with cirrhosis undergoing 6-monthly US surveillance would experience 17.6 surveillance events over their lifetime and have average total surveillance-, cirrhosis-, and HCC-related costs of $136,324 (2023 AUD), a 5.6% increase compared with to those who do not undergo surveillance, with similar increases with the addition of AFP.

**Table 2:** Health outcomes and cost-effectiveness of routine ultrasound surveillance for detection of HCC, with and without alpha-fetoprotein.

Outcome	No Intervention	Six-Monthly US	Six-Monthly US and AFP
HCC incidence per 100,000 persons	21,541	—	—
Proportion of HCC diagnosed at early stage, %^a^	47.0	79.6	81.3
HCC mortality per 100,000 persons	14,918	11,680	11,504
Reduction v. no surveillance, %	—	21.70	22.90
Lifetime liver care costs per 100,000 persons (billions)^b^	$12.91	$13.63	$13.64
Increase v. no surveillance, %	—	5.60	5.60
Mean liver costs per 100,000 persons (billions, discounted)	$8.30	$8.89	$8.99
Quality-adjusted life-years per 100,000 persons	865,630	919,300	922,300
Increase v. no surveillance, %	—	6.20	6.50
Mean QALYs per 100,000 persons (discounted)	472,140	492,156	493,371
Increase v. no surveillance, %	—	4.24	4.50
Incremental cost-effectiveness ratio	—	$28,423/QALY	$79,042/QALY

AFP, alpha-fetoprotein; HCC, hepatocellular carcinoma; QALYs, quality-adjusted life-years; US, ultrasound.

a Barcelona Clinic Liver Cancer stage 0/A.

b Included costs associated with cirrhosis care, routine surveillance, HCC diagnosis, HCC treatment, and costs associated with death. See Appendix Table 6.

The incremental cost-effectiveness of HCC surveillance with 6-monthly US would be $28,423/QALY, below the $50,000/QALY indicative WTP threshold. This indicates that surveillance using 6-monthly US would be cost-effective. Six-monthly surveillance with US and AFP would not be incrementally cost-effective against the $50,000/QALY WTP threshold, with an ICER of $79,042/QALY.

#### Sensitivity analyses

**Figure 3:**
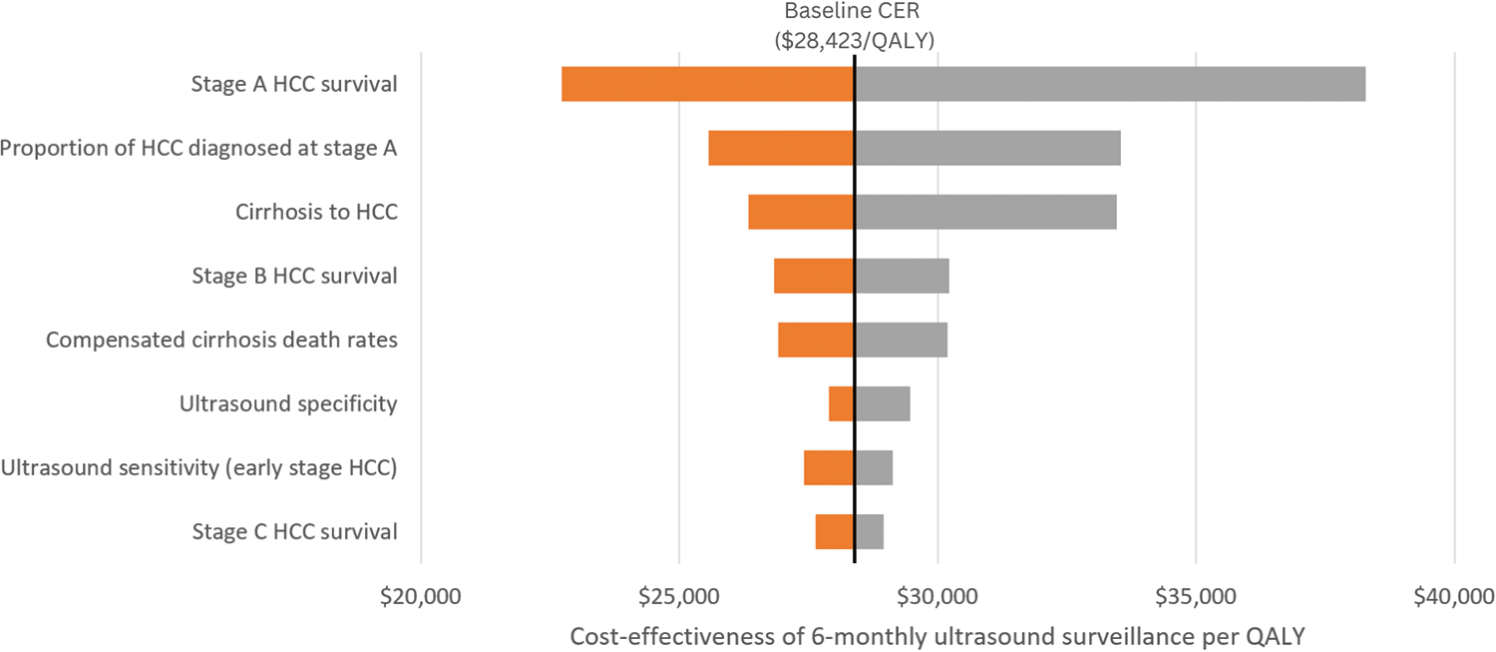
One-way sensitivity analysis: impact of key model parameters on the incremental cost-effectiveness of 6-monthly HCC surveillance with ultrasound versus no surveillance. Parameter ranges for each parameter are included in Appendix A. The parameters with the largest impact are shown. Further sensitivity analyses on the impact of survival and treatment cost parameters by stage are shown in Appendix B. CER, cost-effectiveness ratio; HCC, hepatocellular carcinoma; QALY, quality-adjusted life-year.

The 1-way sensitivity analysis found that the model results were robust to the key health parameters and were most sensitive to parameters around early-stage (BCLC A/0) HCC diagnosis and survival. The parameter ranges were based on the distributions shown in Appendix A. The parameters with the greatest impact on cost-effectiveness are shown in Figure 3. In all cases, the ICER remained below the $50,000/QALY WTP threshold.

**Figure 4:**
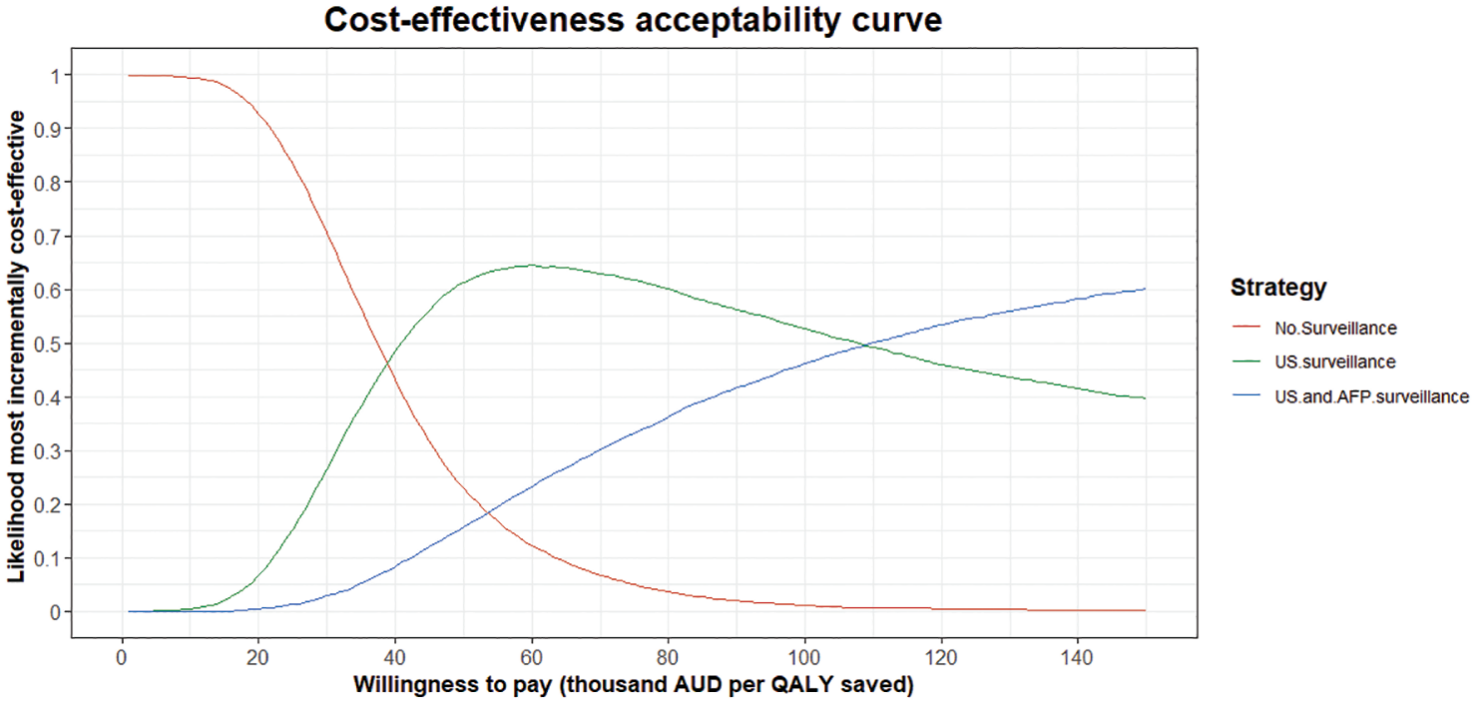
Probabilistic sensitivity analysis: the incremental cost-effectiveness acceptability curve showing the likelihood that no surveillance, 6-monthly ultrasound surveillance, or 6-monthly ultrasound and alpha-fetoprotein surveillance are the most incrementally cost-effective option at a range of willingness-to-pay thresholds, based on the proportion of the 100,000 simulations. AFP, alpha-fetoprotein; QALY, quality-adjusted life-year; US, ultrasound; AUD, Australian dollars.

In the probabilistic sensitivity analysis (Figure 4), at the $50,000/QALY WTP threshold, 6-monthly US without AFP was the most incrementally cost-effective intervention in 61.2% of simulations, 6-monthly US with AFP was the most incrementally cost-effective intervention in 15.7% of simulations, and in 23.0% of simulations neither intervention was incrementally cost-effective.

Lower discount rates led to improved cost-effectiveness for surveillance (Appendix Table 7). No discounting (0%) led to an ICER of $11,007/QALY for US alone and $37,568 for US with AFP, under the $50,000/QALY WTP threshold. Higher discount rates led to lower cost-effectiveness for surveillance, increasing to $39,446 and $103,354 ICERs for US and US with AFP, respectively, at a 7% discount rate.

The findings of the 2-way sensitivity on the impact of HCC treatment costs and survival rates by age are shown in Appendix Figure 1. We found that the cost-effectiveness ratio for 6-monthly US surveillance had a strong positive correlation with higher early (BCLC 0/A) stage treatment costs and lower early-stage 5-y survival, indicating that mortality improvements and cost savings in stage 0/A HCC would improve the cost-effectiveness of surveillance, while additional costs and/or worsened survival would increase the cost-effectiveness. A relative 50% increase in stage 0/A survival would improve the ICER of 6-monthly US surveillance from $28,423/QALY to $17,456/QALY. A 50% decrease in stage 0/A treatment costs would similarly improve the ICER to $18,379/QALY. A 50% increase in stage 0/A treatment costs would increase the ICER to $47,781/QALY, significantly increasing the ICER while remaining below the $50,000/QALY WTP threshold.

A less strong correlation was found between stage B and C treatment costs and survival and cost-effectiveness, with higher costs and lower survival leading to improved cost-effectiveness. This is due to the main benefit of surveillance being diagnosis at stage 0/A rather than later stages. The outcomes were insensitive to changes in stage D treatment costs or effectiveness.

**Figure 5:**
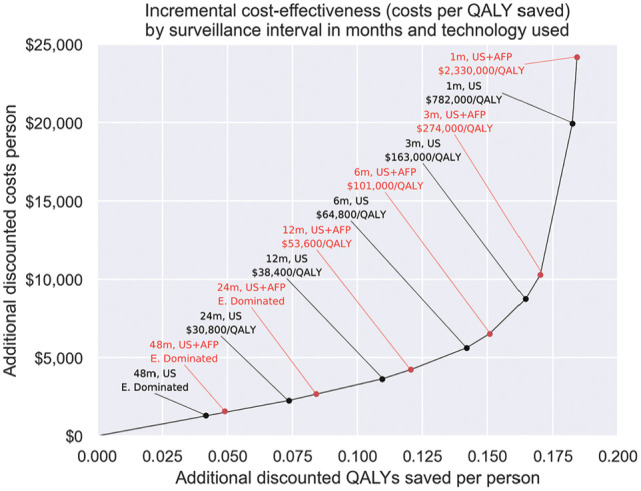
Discounted costs versus discounted QALYs saved for routine US surveillance with or without AFP by surveillance interval in months. Scenarios not on the cost-effectiveness frontier are extended dominated and marked as E. Dominated. AFP, alpha-fetoprotein; QALY, quality-adjusted life-year; US, ultrasound.

The impact of alternative surveillance intervals is shown in Figure 5. Shorter surveillance intervals have higher benefits but at correspondingly higher costs. Additional benefits and costs were roughly proportional at intervals longer than 12 mo, while shorter intervals led to diminishing benefits per cost increase. When considering all combinations of US with or without AFP and the corresponding intervals, all US surveillance at intervals 12 mo or longer would have an ICER below the $50,000/QALY WTP threshold, while US and AFP surveillance would have an ICER below the WTP threshold at intervals greater than 12 mo. The most incrementally cost-effective intervention under the $50,000/QALY WTP threshold is 12-monthly US, with an ICER of $38,400/QALY.

**Figure 6:**
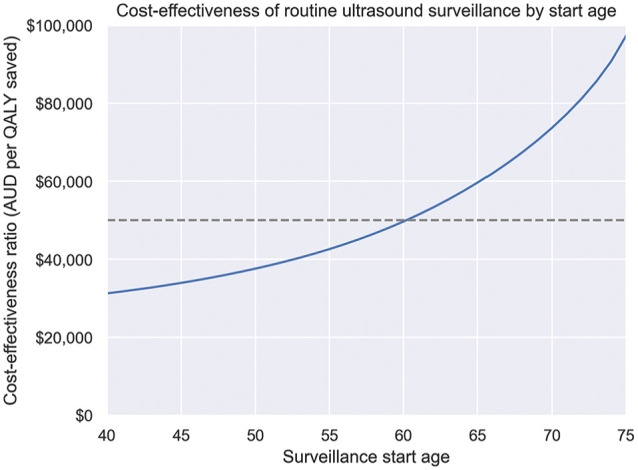
Supplementary analysis: incremental cost-effectiveness ratio for 6-monthly ultrasound surveillance versus no surveillance, by age of cirrhosis diagnosis. The $50,000/QALY willingness-to-pay threshold is indicated. QALY, quality-adjusted life-year; AUD, Australian dollars.

The impact of the age of cirrhosis diagnosis is shown in Figure 6. These results suggest that routine 6-monthly US with AFP is more cost-effective for people at younger ages, due to the higher likelihood of detecting HCC before liver decompensation or death. Routine surveillance was cost-effective under the $50,000/QALY WTP for people who started surveillance at 60 y or earlier.

**Figure 7:**
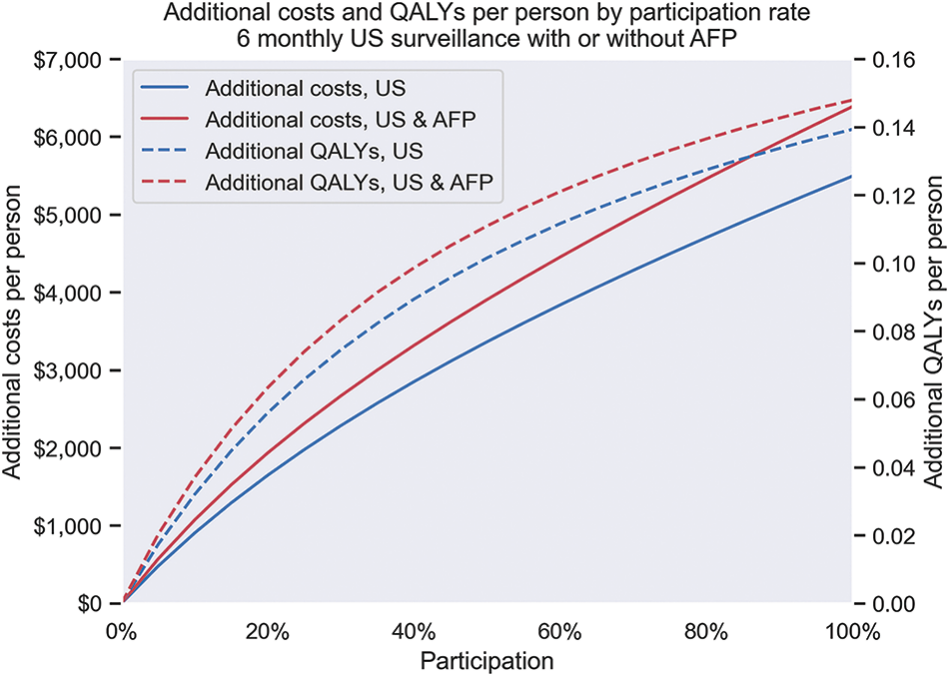
Health benefits and costs associated with 6-monthly surveillance, by level of adherence. Comparator: no surveillance. AFP, alpha-fetoprotein; QALYs, quality-adjusted life-years; US, ultrasound.

The impact of varying adherence rates to 6-monthly US surveillance with or without AFP are shown in Figure 7. Higher levels of adherence were associated with greater health benefits and costs.

## Discussion

We developed the Policy1-Liver model in collaboration with the HCC surveillance guidelines working group and successfully calibrated to the target data. The model estimated that routine HCC surveillance with 6-monthly US would be cost-effective and reduce the likelihood of HCC death. This analysis found that the addition of AFP would have further health benefits and similar costs but may not be incrementally cost-effective. Earlier versions of these findings were included in the 2023 *Clinical Practice Guidelines for HCC Surveillance for People at High Risk in Australia*^8,17^ to support recommendations for the provision of US surveillance. These findings broadly align with similar recent cost-effectiveness studies in Australia^18^ and internationally.^8,27^

The 1-way and probabilistic sensitivity analyses demonstrate that the model findings were robust with respect changes in the parameter values, showing that the uncertainty in the data sources does not qualitatively alter our findings, while also demonstrating the likelihood of incremental cost-effectiveness of surveillance recommendations at a range of WTP thresholds. The sensitivity analysis on the impact of the discount rate found that low discount rates increased perceived cost-effectiveness, reflecting short-term costs of surveillance versus the long-term health benefits. The impact of discounting on the perceived benefits of surveillance should be a consideration for policy makers when comparing to other interventions.

Our additional analysis of the impact of surveillance adherence rates showed that, as expected, higher adherence led to more favorable health outcomes but also additional costs. In the absence of a national program, there are sparse data to inform estimates of liver surveillance adherence in Australia^15,16^; however, a correlation between adherence and HCC survival has been observed.^54^ As surveillance recommendations are more widely adopted, it will be crucial to track longitudinal adherence.

The analysis of the impact of age of cirrhosis diagnosis found that routine surveillance was most cost-effective for people diagnosed at younger ages, who had a lower risk of competing events such as decompensation and non-HCC mortality. In general, the impact of comorbidities and all-cause mortality risk have a significant impact on the cost-effectiveness of surveillance. Where possible, surveillance recommendations should consider an individual’s life expectancy and comorbidities.

The impact of varying surveillance intervals found that longer intervals were more likely to be cost-effective, with US surveillance cost-effective at intervals of 12 mo or longer. We also found that the addition of AFP may allow for similar benefits and costs as US alone at a longer interval, although was this is not always incrementally cost-effective. In the analysis incorporating a wider range of surveillance intervals, 6-monthly surveillance was not incrementally cost-effective compared with the $50,000/QALY WTP threshold, with an ICER of $64,800/QALY for US alone. This indicates a need for further consideration and refinement of surveillance recommendations. Longer surveillance intervals may also be caused by patient noncompliance, delays, resource limitations, or other limitations. Most notably, there is a shortage of sonographers in Australia, which could lead to longer surveillance intervals.^55^ Our findings indicate that surveillance at these longer intervals would still be cost-effective. These findings could assist with future research to optimize surveillance recommendations, particularly in developing patient tailored recommendations and/or recommendations for settings where resources are limited.

One strength of the analysis was the use of the sojourn time density model structure,^30^ which allows for flexible analyses incorporating detailed survival data and flexible time scales. This gives an advantage over Markov models of liver cancer,^7,8^ which may not be able to exploit complex survival data or reflect detailed disease progression^56,57^ or larger microsimulation models that can be computationally expensive and introduce stochasticity. This structure allowed us to use complex survival data directly in our model with minimal recalibration. Having a flexible and arbitrarily small time steps allowed us to compare surveillance intervals of any length and avoid potential biases due to time-scale granularity.^58^ Having a computationally inexpensive model allowed us to run probabilistic sensitivity analyses with hundreds of thousands of parameter sets in a reasonable time frame, fully assessing the potential parameter uncertainties in our model. Having a deterministic modeling approach also means we can assess the impact of rare events without loss of precision, such as in model simulations with very low adherence rates. It is hoped that the advantages of this approach to modeling are of value to other public health practitioners.

This model considers only the Australian population with liver cirrhosis. Liver cirrhosis is typically attributable to chronic hepatitis B, chronic hepatitis C, alcohol-related liver disease, metabolic-associated fatty liver disease, or a combination of these. Each of these groups have differing HCC risks, potentially differing treatment needs^59^ and differing prevalence in the Australian population.^60,61^ Although these groups are included in the aggregate liver cirrhosis population modeled here, they are not modeled explicitly by etiology. Future model expansions to capture these risk groups explicitly will allow more precise cost-effectiveness estimates and recommendations by group, identifying the optimal benefit of health benefits and costs between surveillance technology options and varying intervals.

Another limitation of our model is that treatment is not explicitly modeled by modality; patients are instead modeled based on aggregate survival and costs by stage at diagnosis. Although this is in line with many other models of cancer and surveillance,^23^ detailed models of HCC treatment can provide additional detail, particularly regarding health service utilization.^18^ Treatment in HCC is rapidly evolving; of particular note, sorafenib was included as a treatment option based on the available data to inform the model construction, but as of 2024, new immunotherapies have supplanted sorafenib.^62^ The 2-way sensitivity analysis presented in Appendix Figure 1 shows that improvements in early-stage survival can improve the cost-effectiveness of surveillance, but increased costs would make surveillance less cost-effective while remaining under the $50,000/QALY WTP threshold. Identifying data with sufficient statistical power and time horizons while remaining up to date with new treatments is an ongoing challenge in health economic modeling. Further research is required to assess the impact of new technologies, derive more detailed patient costs, and increase the fidelity of treatment modeling in line with equivalent models in lung cancer.^63^

The current simulation of the influence of age on HCC risk was limited by a lack of data. Currently, the modeled age influences HCC risk through time exposed to cirrhosis and risk of other-cause mortality. Incorporating further targets for the evolving risk of HCC and stage at diagnosis would improve the cost-effectiveness estimates and help refine recommendations. In general, data regarding HCC development and surveillance are limited; for example, differences in estimated stage at diagnosis,^33,64^ with significant differences likely attributable to surveillance adherence and patient cohorts and sensitivity and specificity of US.^34,65^ Australia-specific elicitation of quality-of-life weights in HCC patients would also improve the quality of modeling. The utility weights used here were for a non-Australian cohort but were generally consistent with weights used in other HCC modeling studies.^66^ The utility weights used in this study did not discriminate patients with decompensated liver cirrhosis; although this does not affect our findings, as individuals with decompensation are not recommended surveillance, understanding quality of life in this cohort would improve our understanding of liver disease prevention. As further data are made available, modeling will be updated and refined to help guide interventions, including more complex surveillance algorithms, such as the FIB-4 algorithm recommended by the Asian Pacific Association for the Study of the Liver clinical guidelines,^67^ for people with pre–cirrhotic liver disease. The Policy1-Liver model has been designed for straightforward expansion to these further analyses and updated data.

## Conclusion

There is significant potential for routine HCC surveillance to improve mortality outcomes in Australia. However, any recommendations must account for the costs and the consequences of the lower life expectancy and quality of life for high-risk individuals. A robust and flexible model of liver disease and surveillance was developed to estimate the impact of routine surveillance. We found that 6-monthly routine HCC surveillance with US would be cost-effective but that health and economic outcomes varied with differing adherence, cirrhosis diagnosis age, surveillance intervals, and the use of AFP. These results could inform future surveillance recommendations, including a national screening program for liver cancer in Australia. Further evaluations could provide refinements and extensions of the economic evaluations as more robust data become available to capture detailed patient risk groups.

## Supplemental Material

sj-docx-1-mpp-10.1177_23814683251344962 – Supplemental material for A Health Economic Evaluation of Routine Hepatocellular Carcinoma Surveillance for People with Compensated Cirrhosis to Support Australian Clinical Guidelines Supplemental material, sj-docx-1-mpp-10.1177_23814683251344962 for A Health Economic Evaluation of Routine Hepatocellular Carcinoma Surveillance for People with Compensated Cirrhosis to Support Australian Clinical Guidelines by Joachim Worthington, Emily He, Michael Caruana, Stephen Wade, Barbara de Graaff, Anh Le Tuan Nguyen, Jacob George, Karen Canfell and Eleonora Feletto in MDM Policy & Practice
