# Supplementary material for: A Health Economic Evaluation of Routine Hepatocellular Carcinoma Surveillance for People with Compensated Cirrhosis to Support Australian Clinical Guidelines
Source: MDM Policy Pract. 2025 Jun 26;10(1):23814683251344962. doi: 10.1177/23814683251344962 (PMC12202943; doi:10.1177/23814683251344962)
Supplement: sj-docx-1-mpp-10.1177_23814683251344962 – Supplemental material for A Health Economic Evaluation of Routine Hepatocellular Carcinoma Surveillance for People with Compensated Cirrhosis to Support Australian Clinical Guidelines [file sj-docx-1-mpp-10.1177_23814683251344962.docx]

## A health economic evaluation of routine hepatocellular carcinoma surveillance for people with compensated cirrhosis to support Australian clinical guidelines: Supplementary Materials

## Appendix A: Model Parameters

Appendix Table 1 - Calibrated precancer natural history parameters for the model of HCC. $\tau$ is the sojourn time spent in a particular disease state.

| **Description** | **Model value** | **Target**  **(95% CI)** | **Source and notes** |
| --- | --- | --- | --- |
| **Cirrhosis Decompensation** | | | |
| Hazard rate | $\lambda=\left\{ \begin{aligned} 0.0204\tau+0.0106 for \tau<6 \\ 0.0554 \text{for} \tau\geq6 \end{aligned} \right.$ |  | Calibrated to reproduce published rates (see below).* |
| Relative risk for probabilistic sensitivity analysis | $RR\sim\text{Lognormal(}\mu=\text{-}{0.0213,\sigma^{2}=0.117}^{2}$) |  | Distribution of relative risk vs baseline hazard**.^1^ |
| *Calibration target:* 1 year probability | 1.03% | 1.59%  (0.17-3.01%) | ^1^ |
| *Calibration target:* 10 year probability | 34.5% | 33.7%  (28.4-39.1%) |  |
| **HCC development** | | | |
| Hazard rate | $\lambda=0.0102\tau^{1.377}$ |  | Calibrated to reproduce published rates (see below).* |
| Relative risk for probabilistic sensitivity analysis | $RR\sim\text{Lognormal}\text{(}\mu=\text{-}{0.00171,\sigma^{2}=0.158}^{2}$*)* |  | Distribution of relative risk vs baseline hazard**.^1^ |
| *Calibration target:* 1 year probability | 0.99% | 1.53%  (0.14-2.93% ) | ^1^ |
| *Calibration target:* 10 year probability | 15.2% | 15.6%  (11.5-19.7% ) |  |
| **Death (compensated cirrhosis)** | | | |
| Hazard rate | $\lambda=0.0336\tau^{-0.2379}$ |  | Calibrated to reproduce published rates (see below).* |
| Relative risk for probabilistic sensitivity analysis | $RR\sim\text{Lognormal}\text{(}\mu={-0.0254,\sigma^{2}=0.158}^{2}$) |  | Distribution of relative risk vs baseline hazard**.^1^ |
| *Calibration target:* 1 year probability | 4.72% | 3.97%  (2.62-5.31% ) | ^2^ |
| *Calibration target:* 10 year probability | 39.4% | 39.4%  (36.1-42.8% ) |  |
| **Death (decompensated cirrhosis)** | | | |
| Hazard rate | $\lambda=\left( 0.1539\tau-0.4865 \right)^{2}+0.2103$ |  | Calibrated to reproduce published rates (see below).* |
| Relative risk for probabilistic sensitivity analysis | $RR\sim\text{Lognormal}\text{(}\mu={0.0478,\sigma^{2}=0.0895}^{2}$) |  | Distribution of relative risk vs baseline hazard**.^1^ |
| *Calibration target:* 1 year probability | 37.8% | 38.3%  (35.0-41.6% ) | ^2^ |
| *Calibration target:* 10 year probability | 90.6% | 91.1%  (89.2-93.0% ) |  |

* The full calibration includes a range of target data points not shown here; see e.g. **Error! Reference source not found.**.

** Relative risk is a constant multiplier to the relative baseline hazard, drawn from the given distribution.

Appendix Table 2 - Calibrated model parameters relating to the progression, detection, and survival of HCC.

| **Description** | **Model value** | **Source / Notes** |
| --- | --- | --- |
| **Early (Stage 0/A), undiagnosed** | | Progression and detection values used as calibration targets for stage at diagnosis, with and without regular ultrasound surveillance. Sensitivity analysis is the distribution of relative risk vs baseline hazard. |
| Stage progression hazard | 0.2049 |  |
| Relative risk for probabilistic sensitivity analysis* | $RR\sim\text{Lognormal}\text{(}\mu=0.008243{,\sigma^{2}=0.1852}^{2}$*)* |  |
| Detection hazard | 0.2091 |  |
| Relative risk for probabilistic sensitivity analysis* | $RR\sim\text{Lognormal}\text{(}\mu=-0.000217{,\sigma^{2}=0.1826}^{2}$*)* |  |
| **Intermediate (Stage B), undiagnosed** | |  |
| Stage progression hazard | 1.0259 |  |
| Relative risk for probabilistic sensitivity analysis* | $RR\sim\text{Lognormal}\text{(}\mu=-0.01844{,\sigma^{2}=0.3365}^{2}$*)* |  |
| Detection hazard | 1.1727 |  |
| Relative risk for probabilistic sensitivity analysis* | $RR\sim\text{Lognormal}\text{(}\mu=0.0001390{,\sigma^{2}=0.3282}^{2}$*)* |  |
| **Late (Stage C/D), undiagnosed** | |  |
| Detection hazard | 1.4887 |  |
| Relative risk for probabilistic sensitivity analysis* | $RR\sim\text{Lognormal}\text{(}\mu=-0.001311{,\sigma^{2}=0.05120}^{2}$*)* |  |
| **Five-year survival probability** | | |
| Local spread (Stage 0/A/B) | 47.7% | **Target**: 47.7%.^3^ |
| Regional/Distant Spread (Stage C/D) | 20.6% | **Target**: 20.6%.^3^ |
| **Hazard ratio for five-year survival by stage** | | |
| Stage B vs Stage 0/A | 0.508 | **Target**: 0.508.^4^ |
| Stage D vs Stage C | 0.841 | **Target**: 0.841.^4^ |
| **Relative risk of survival for probabilistic sensitivity analysis*** | | |
| Stage 0/A | $RR\sim\text{Lognormal(}\mu=-0.02333{,\sigma^{2}=0.1487}^{2}$) | ^4^ |
| Stage B | $RR\sim\text{Lognormal(}\mu=-0.02702{,\sigma^{2}=0.1076}^{2}$) |  |
| Stage C | $RR\sim\text{Lognormal(}\mu=-0.1286{,\sigma^{2}=0.08124}^{2}$) |  |
| Stage D | $RR\sim\text{Lognormal(}\mu=-0.07029{,\sigma^{2}=0.1246}^{2}$) |  |

* Relative risk is a constant multiplier to the relative baseline hazard, drawn from the given distribution.

Appendix Table 3 - Calibrated model parameters relating to surveillance sensitivity and specificity. US: ultrasound. AFP: alpha-fetoprotein.

| **Description** | **Model value**  **Distribution for probabilistic sensitivity analysis** | **Target**  **95% CI** | **Source and notes** |
| --- | --- | --- | --- |
| US sensitivity,  stage 0/A HCC | 53%  $\text{Beta(}\alpha=15.87,\beta=14.23$) | 53%  35-70% | ^5^ |
| US sensitivity,  stage B/C/D HCC | 84%  $\text{Beta(}\alpha=29.03,\beta=6.735$) | 84%  67-92% |  |
| US specificity | 91%  $\text{Beta(}\alpha=186.8,\beta=19.86$) | 91%  86-94% |  |
| US & AFP sensitivity,  stage 0/A HCC | 63%  $\text{Beta(}\alpha=29.69,\beta=18.22$) | 63%  48-75% |  |
| US & AFP sensitivity,  stage B/C/D HCC | 97%  $\text{Beta(}\alpha=84.54,\beta=3.530$) | 97%  91-99% |  |
| US & AFP specificity | 84%  $\text{Beta(}\alpha=121.2,\beta=24.07$) | 84%  77-89% |  |

Appendix Table 4 – Calibration targets for HCC stage at detection with and without surveillance. Outcomes based on six-monthly surveillance, with BCLC stage recorded at diagnosis and any post-diagnosis stage progression excluded. US: ultrasound. AFP: alpha-fetoprotein.

| **Description** | **Model outcome** | **Target**  **95% CI** | **Source and notes** |
| --- | --- | --- | --- |
| Stage 0/A HCC proportion, no surveillance | 47% | 47%  39-55% | Calibration target ^6^ |
| Stage B HCC proportion, no surveillance | 24% | 24%  17-31% |  |
| Stage C/D HCC proportion, no surveillance | 29% | 29%  18-40% |  |
| Stage 0/A HCC proportion, US surveillance | 47% | 47%  39-55% |  |
| Stage B HCC proportion, US surveillance | 24% | 24%  17-31% |  |
| Stage C/D HCC proportion, US surveillance | 29% | 29%  18-40% |  |
| Stage 0/A HCC proportion, US & AFP surveillance | 83% | - | Model outcomes based on test characteristics in Appendix Table 3. |
| Stage B HCC proportion, US & AFP surveillance | 7% | - |  |
| Stage C/D HCC proportion, US & AFP surveillance | 10% | - |  |

Appendix Table 5 – Treatment modality proportions for diagnosis and treatment of HCC. Treatments are listed sequentially, with primary treatment modality listed first. CT: computed tomography. MRI: magnetic resonance imaging. TACE: transarterial chemoembolization.

| **Primary treatment** | **Secondary treatment(s)** | **Proportion** | **Distribution*** | **Source and notes** |
| --- | --- | --- | --- | --- |
| CT | - | 80% | $\text{Beta}\left( \alpha=164.0,\beta=23.94 \right)$ | ^7^. Includes attempted diagnosis after false positive surveillance event. Biopsy used to assess inconclusive CT or MRI |
| MRI | - | 20% | $\text{Beta}\left( \alpha=23.12,\beta=161.90 \right)$ |  |
| Biopsy | - | 10% | $\text{Beta}\left( \alpha=18.13,\beta=165.80 \right)$ |  |
| **Early stage HCC (stage 0/A) treatment** | | | | Based on outcomes and confidence intervals reported in ^8^. Used to calculate treatment costs. |
| Transplant |  | 19.00% | $\text{Beta}\left( \alpha=12.86,\beta=54.81 \right)$ |  |
| Resection | - | 13.80% | $\text{Beta}\left( \alpha=3.05,\beta=19.04 \right)$ |  |
| Ablation | - | 25.60% | $\text{Beta}\left( \alpha=16.48,\beta=47.91 \right)$ |  |
| TACE | - | 34.80% | $\text{Beta}\left( \alpha=21.33,\beta=39.96 \right)$ |  |
| Resection | Ablation/TACE, then sorafenib | 3.40% | $\text{Beta}\left( \alpha=3.05,\beta=86.61 \right)$ |  |
| Ablation | Sorafenib | 1.50% | $\text{Beta}\left( \alpha=1.45,\beta=95.51 \right)$ |  |
| TACE | Sorafenib | 2.00% | $\text{Beta}\left( \alpha=1.90,\beta=93.19 \right)$ |  |
| **Intermediate stage HCC (stage B) treatment** | | | |  |
| Transplant | - | 8.30% | $\text{Beta}\left( \alpha=1.57,\beta=17.37 \right)$ |  |
| Resection | - | 8.30% | $\text{Beta}\left( \alpha=1.57,\beta=17.37 \right)$ |  |
| Ablation | - | 17.70% | $\text{Beta}\left( \alpha=2.77,\beta=12.90 \right)$ |  |
| TACE | - | 24.00% | $\text{Beta}\left( \alpha=3.44,\beta=10.89 \right)$ |  |
| Ablation | Sorafenib | 14.10% | $\text{Beta}\left( \alpha=2.38,\beta=14.52 \right)$ |  |
| TACE | Sorafenib | 19.20% | $\text{Beta}\left( \alpha=2.95,\beta=12.40 \right)$ |  |
| Resection | Sorafenib | 8.30% | $\text{Beta}\left( \alpha=1.57,\beta=17.37 \right)$ |  |
| **Late stage HCC (stage C/D) treatment** | | | |  |
| Ablation | - | 3.40% | $\text{Beta}\left( \alpha=1.44,\beta=41.00 \right)$ |  |
| TACE | - | 4.60% | $\text{Beta}\left( \alpha=1.87,\beta=38.68 \right)$ |  |
| Ablation | Sorafenib | 6.80% | $\text{Beta}\left( \alpha=2.55,\beta=34.93 \right)$ |  |
| TACE | Sorafenib | 9.20% | $\text{Beta}\left( \alpha=3.24,\beta=31.99 \right)$ |  |
| Ablation | Palliation | 11.90% | $\text{Beta}\left( \alpha=4.02,\beta=29.79 \right)$ |  |
| TACE | Palliation | 16.10% | $\text{Beta}\left( \alpha=5.14,\beta=26.77 \right)$ |  |
| Sorafenib | Palliation | 16.00% | $\text{Beta}\left( \alpha=5.00,\beta=26.25 \right)$ |  |
| Palliation | - | 32.00% | $\text{Beta}\left( \alpha=8.68,\beta=18.44 \right)$ |  |

*Distribution for probabilistic sensitivity analysis.

Appendix Table 6 – Modelled cost and utility parameters.

| **Description** | **Value** | **Distribution*** | **Source and notes** |
| --- | --- | --- | --- |
| **Annual cirrhosis care costs** | | | ^9^ |
| Compensated | $4,713 | $\text{Lognormal}\left( \mu=8.047,\sigma=0.527 \right)$ |  |
| Decompensated | $22,701 | $\text{Lognormal}\left( \mu=9.861,\sigma=0.306 \right)$ |  |
| **Surveillance-related costs** | | | Medicare benefits schedule.^10^ Calibrated to 20% increase and decrease in costs, in the absence of other targets. |
| Ultrasound | $115.75 | $\text{Lognormal}\left( \mu=4.731,\sigma=0.104 \right)$ |  |
| AFP | $24.35 | $\text{Lognormal}\left( \mu=3.172,\sigma=0.104 \right)$ |  |
| GP Visit | $39.75 | $\text{Lognormal}\left( \mu=3.662,\sigma=0.104 \right)$ |  |
| CT (diagnostic) | $499.50 | $\text{Lognormal}\left( \mu=6.193,\sigma=0.104 \right)$ |  |
| MRI (diagnostic) | $558.80 | $\text{Lognormal}\left( \mu=6.305,\sigma=0.103 \right)$ |  |
| Liver biopsy (diagnostic) | $377.2 | $\text{Lognormal}\left( \mu=5.912,\sigma=0.104 \right)$ |  |
| **Treatment-related costs** | | | ^11^ Proportion of patients allocated to RFA/MWA/PEI based on the proportions reported in Hong et al.^11^ TACE includes TACE with cisplantin, TACE with doxorubicin, and SIRT. |
| Liver transplant | $320,107 | $\text{Lognormal}\left( \mu=12.731,\sigma=0.367 \right)$ |  |
| Liver resection | $73,310 | $\text{Lognormal}\left( \mu=11.09,\sigma=0.556 \right)$ |  |
| Ablation (RFA/MWA/PEI) | $94,611 | $\text{Lognormal}\left( \mu=11.292,\sigma=1.168 \right)$ |  |
| TACE4 | $76,482 | $\text{Lognormal}\left( \mu=11.252,\sigma=0.85 \right)$ |  |
| Sorafenib | $42,338 | $\text{Lognormal}\left( \mu=10.498,\sigma=0.756 \right)$ |  |
| Palliation | $45,084 | $\text{Lognormal}\left( \mu=10.561,\sigma=0.622 \right)$ |  |
| **End-of-life costs** | | | ^12^ |
| Death from cancer | $44,945 | $\text{Lognormal}\left( \mu=10.713,\sigma=0.011 \right)$ |  |
| Death from other causes | $31,513 | $\text{Lognormal}\left( \mu=10.358,\sigma=0.012 \right)$ |  |
| **Health state utility values** | | | |
| Liver cirrhosis | 0.56 | $\text{Beta}(\alpha=74,\beta=58)$ | ^13^ |
| HCC with non-transplant surgery | 0.61 | $\text{Beta}(\alpha=66,\beta=42)$ |  |
| HCC with non-surgical treatment | 0.64 | $\text{Beta}(\alpha=60,\beta=34)$ |  |
| HCC with liver transplant | 0.67 | $\text{Beta}(\alpha=55,\beta=27)$ |  |
| HCC with palliation | 0.39 | $\text{Beta}(\alpha=103,\beta=161)$ |  |

*Distribution for probabilistic sensitivity analysis.

** Calibrated to 20% increase and decrease in costs, in the absence of other targets.

## Appendix B: Additional sensitivity analyses

#### Sensitivity analysis on treatment costs and survival by stage

Appendix Figure 1 –Two-way sensitivity analysis on the impact of alternative survival and treatment cost parameters by stage on cost-effectiveness of six-monthly ultrasound surveillance vs no surveillance.


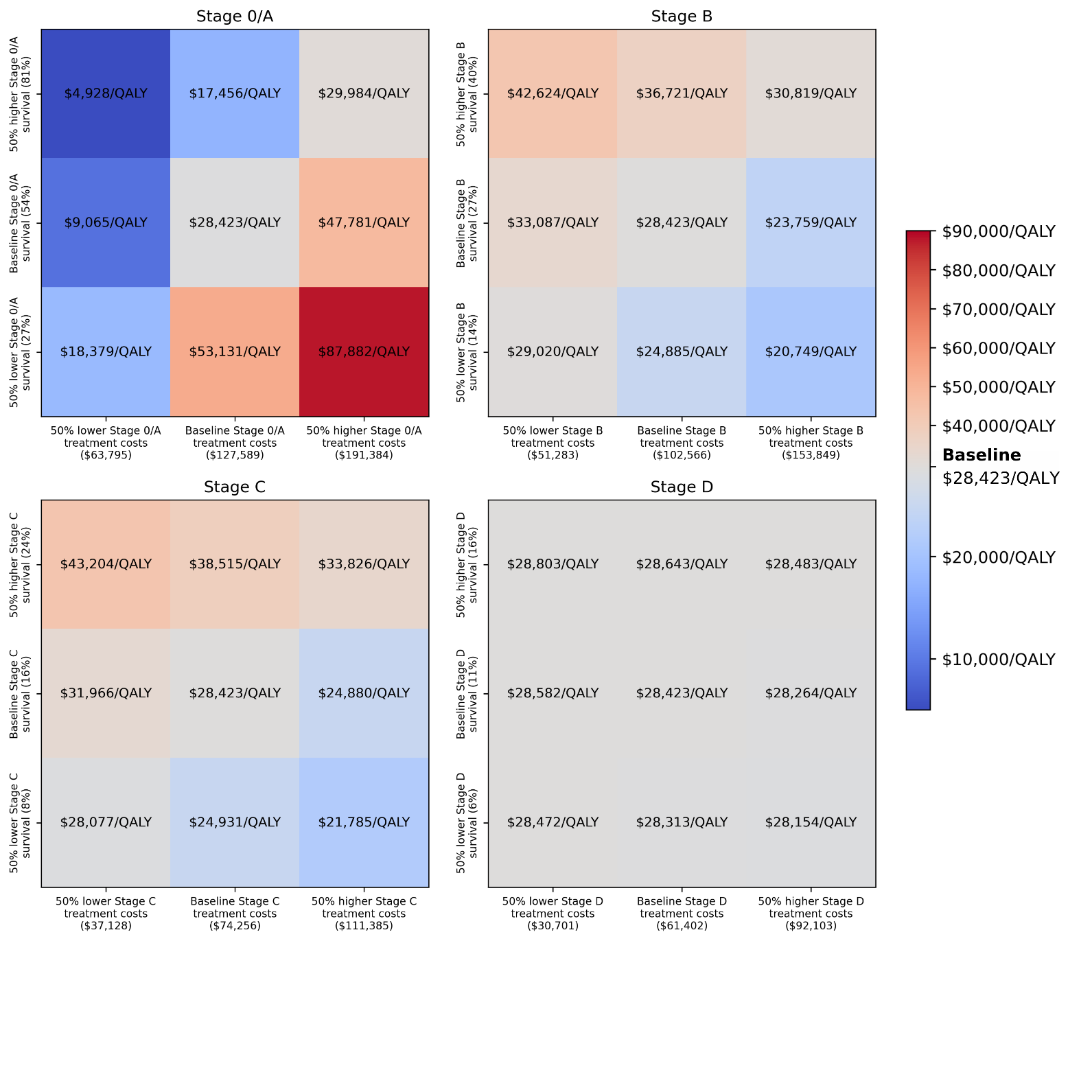


#### Sensitivity analysis on alternative discount rates

Appendix Table 7 - Sensitivity analysis on the discount rate used in the economic analysis. Discounting was applied to both QALYs and costs from surveillance start time, with six-monthly surveillance.

|  | **No Surveillance** | | **6 monthly US surveillance** | | | **6 monthly US surveillance with AFP** | | |
| --- | --- | --- | --- | --- | --- | --- | --- | --- |
| **Discount**  **rate** | **Discounted costs per person** | **Discounted QALYs per person** | **Discounted costs per person** | **Discounted QALYs per person** | **ICER (vs no surveillance)** | **Discounted costs per person** | **Discounted QALYs per person** | **ICER (vs US alone)** |
| **0%** | $129,000 | 7.356 | $136,000 | 7.915 | $11,007 | $137,000 | 7.947 | $37,568 |
| **1.5%** | $111,000 | 6.313 | $118,000 | 6.715 | $14,955 | $119,000 | 6.738 | $47,541 |
| **3%** | $97,400 | 5.518 | $104,000 | 5.813 | $19,931 | $105,000 | 5.831 | $59,516 |
| **5%** | $83,000 | 4.721 | $88,900 | 4.922 | $28,423 | $89,900 | 4.934 | $79,042 |
| **7%** | $72,000 | 4.126 | $77,600 | 4.266 | $39,446 | $78,600 | 4.274 | $103,354 |

## Appendix C: CHEERS Checklist

See Husereau et al for further details.^14^

Appendix Table 8 - CHEERS checklist for the model of HCC and surveillance.

| **Topic** | **No.** | **Item** | **Location where item is reported** |
| --- | --- | --- | --- |
| **Title** | | | |
| **Title** | 1 | Identify the study as an economic evaluation and specify the interventions being compared. | Title, Page 1 |
| **Abstract** | | | |
| **Abstract** | 2 | Provide a structured summary that highlights context, key methods, results, and alternative analyses. | Abstract, Page 1 |
| **Introduction** | | | |
| **Background and objectives** | 3 | Give the context for the study, the study question, and its practical relevance for decision making in policy or practice. | Introduction, Page 3 |
| **Methods** | | | |
| **Health economic analysis plan** | 4 | Indicate whether a health economic analysis plan was developed and where available. | N/A |
| **Study population** | 5 | Describe characteristics of the study population (such as age range, demographics, socioeconomic, or clinical characteristics). | Methods, page 5 |
| **Setting and location** | 6 | Provide relevant contextual information that may influence findings. | Methods, page 5-6 |
| **Comparators** | 7 | Describe the interventions or strategies being compared and why chosen. | Methods, pages 4, 7 |
| **Perspective** | 8 | State the perspective(s) adopted by the study and why chosen. | Methods, page 6 |
| **Time horizon** | 9 | State the time horizon for the study and why appropriate. | Methods, page 6 |
| **Discount rate** | 10 | Report the discount rate(s) and reason chosen. | Methods, page 7 |
| **Selection of outcomes** | 11 | Describe what outcomes were used as the measure(s) of benefit(s) and harm(s). | Methods, page 5-6 |
| **Measurement of outcomes** | 12 | Describe how outcomes used to capture benefit(s) and harm(s) were measured. | N/A |
| **Valuation of outcomes** | 13 | Describe the population and methods used to measure and value outcomes. | Methods, page 5 |
| **Measurement and valuation of resources and costs** | 14 | Describe how costs were valued. | Methods, page 6-7 |
| **Currency, price date, and conversion** | 15 | Report the dates of the estimated resource quantities and unit costs, plus the currency and year of conversion. | Methods, page 6 |
| **Rationale and description of model** | 16 | If modelling is used, describe in detail and why used. Report if the model is publicly available and where it can be accessed. | Methods, page 4-6 and Results page 8 |
| **Analytics and assumptions** | 17 | Describe any methods for analysing or statistically transforming data, any extrapolation methods, and approaches for validating any model used. | Methods, page 5. Appendix A. |
| **Characterising heterogeneity** | 18 | Describe any methods used for estimating how the results of the study vary for subgroups. | N/A |
| **Characterising distributional effects** | 19 | Describe how impacts are distributed across different individuals or adjustments made to reflect priority populations. | N/A |
| **Characterising uncertainty** | 20 | Describe methods to characterise any sources of uncertainty in the analysis. | Methods, page 5 |
| **Approach to engagement with patients and others affected by the study** | 21 | Describe any approaches to engage patients or service recipients, the general public, communities, or stakeholders (such as clinicians or payers) in the design of the study. | Introduction, page 3 |
| **Results** | | | |
| **Study parameters** | 22 | Report all analytic inputs (such as values, ranges, references) including uncertainty or distributional assumptions. | Appendix A |
| **Summary of main results** | 23 | Report the mean values for the main categories of costs and outcomes of interest and summarise them in the most appropriate overall measure. | Results, Table 1 |
| **Effect of uncertainty** | 24 | Describe how uncertainty about analytic judgments, inputs, or projections affect findings. Report the effect of choice of discount rate and time horizon, if applicable. | Results, page 8-10. Appendix Table 7 |
| **Effect of engagement with patients and others affected by the study** | 25 | Report on any difference patient/service recipient, general public, community, or stakeholder involvement made to the approach or findings of the study | Introduction, page 3 |
| **Discussion** | | | |
| **Study findings, limitations, generalisability, and current knowledge** | 26 | Report key findings, limitations, ethical or equity considerations not captured, and how these could affect patients, policy, or practice. | Discussion and conclusion, page 10-13 |
| **Other relevant information** | | | |
| **Source of funding** | 27 | Describe how the study was funded and any role of the funder in the identification, design, conduct, and reporting of the analysis | Title page |
| **Conflicts of interest** | 28 | Report authors conflicts of interest according to journal or International Committee of Medical Journal Editors requirements. | Title page |

## References

1. Vilar-Gomez E, Calzadilla-Bertot L, Wong VW-S, et al. Fibrosis severity as a determinant of cause-specific mortality in patients with advanced nonalcoholic fatty liver disease: a multi-national cohort study. *Gastroenterology* 2018; 155: 443–457.

2. D’Amico G, Garcia-Tsao G, Pagliaro L. Natural history and prognostic indicators of survival in cirrhosis: a systematic review of 118 studies. *J Hepatol* 2006; 44: 217–231.

3. NSW Cancer Registry. *Data request: Hepatocellular carcinoma (coded as C22.0 or C22.9) incidence and mortality)*. Private communication; unpublished., 31 August 2021.

4. Haq MI, Drake TM, Goh TL, et al. Effect of hepatocellular carcinoma surveillance programmes on overall survival in a mixed cirrhotic UK population: a prospective, longitudinal cohort study. *J Clin Med* 2021; 10: 2770.

5. Tzartzeva K, Obi J, Rich NE, et al. Surveillance imaging and alpha fetoprotein for early detection of hepatocellular carcinoma in patients with cirrhosis: a meta-analysis. *Gastroenterology* 2018; 154: 1706–1718.

6. Huang Y, Wallace MC, Adams LA, et al. Rate of nonsurveillance and advanced hepatocellular carcinoma at diagnosis in chronic liver disease. *J Clin Gastroenterol* 2018; 52: 551–556.

7. Nguyen ALT, Si L, Lubel JS, et al. Hepatocellular carcinoma surveillance based on the Australian Consensus Guidelines: a health economic modelling study. *BMC Health Serv Res* 2023; 23: 378.

8. Cheng R. *Novel Biomarkers of Hepatocellular Carcinoma*. PhD Thesis, The University of Sydney, http://hdl.handle.net/2123/20294 (2018).

9. Xiao Y, Howell J, van Gemert C, et al. Enhancing the hepatitis B care cascade in Australia: a cost-effectiveness model. *J Viral Hepat* 2020; 27: 526–536.

10. Department of Health, Australian Government. *MBS Online: Medicare Benefits Schedule. [homepage on the internet]*. Canberra, ACT: Commonwealth of Australia, http://www.mbsonline.gov.au/internet/mbsonline/publishing.nsf/Content/Home.

11. Hong TP. *An Australian population-based study of the incidence and outcomes of hepatocellular carcinoma: the hepatomas of melbourne epidemiological research (homer) study*. PhD Thesis, University of Melbourne, http://hdl.handle.net/11343/225659 (2019).

12. Reeve R, Srasuebkul P, Langton JM, et al. Health care use and costs at the end of life: a comparison of elderly Australian decedents with and without a cancer history. *BMC Palliat Care* 2018; 17: 1–10.

13. Ock M, Lim SY, Lee H-J, et al. Estimation of utility weights for major liver diseases according to disease severity in Korea. *BMC Gastroenterol* 2017; 17: 103.

14. Husereau D, Drummond M, Augustovski F, et al. Consolidated Health Economic Evaluation Reporting Standards 2022 (CHEERS 2022) Explanation and Elaboration: A Report of the ISPOR CHEERS II Good Practices Task Force. *Value Health J Int Soc Pharmacoeconomics Outcomes Res*; 25. Epub ahead of print January 2022. DOI: 10.1016/j.jval.2021.10.008.
